# Prediction of hyper-bilirubinemia in healthy neonates using umbilical cord blood bilirubin

**DOI:** 10.6026/973206300210906

**Published:** 2025-05-31

**Authors:** Madhav Shridharrao Kadam, Mohit Garg, Seema Sutay, Sandeep Singh Matreja, Priyadarshini Rangari, Amit Rangari

**Affiliations:** 1 Department of Biochemistry, Parbhani Medical College and RP Hospital Research Institute, Maharashtra, India; 2 Department of General Medicine, Nandkumar Singh Chouhan Government Medical College, Khandwa, Madhya Pradesh, India; 3 Department of Forensic Medicine and Toxicology, Nandkumar Singh Chouhan Government Medical College, Khandwa, Madhya Pradesh, India; 4 Department of Pathology, Nandkumar Singh Chouhan Government Medical College, Khandwa, Madhya Pradesh, India; 5 Department of Dentistry, Nandkumar Singh Chouhan Government Medical College, Khandwa, Madhya Pradesh, India; 6 Department of Microbiology, Nandkumar Singh Chouhan Government Medical College, Khandwa, Madhya Pradesh, India

**Keywords:** Cord blood bilirubin, neonates, neonatal hyper-bilirubinemia, phototherapy, serum total bilirubin

## Abstract

Jaundice is a clinical condition characterized by transient deficiency of bilirubin conjugation, leading to neonatal hyper-bilirubinemia. Therefore, it is of interest to assess cord blood bilirubin at birth as a predictor of neonatal hyper-bilirubinemia needing phototherapy in full-term neonates. In 220 neonates with hyper-bilirubinemia needing phototherapy were assigned as cases and without hyper-bilirubinemia as controls to assess association in serum total bilirubin and cord blood bilirubin were assessed after identifying the cut-off level of cord blood bilirubin. The mean cord blood bilirubin level was 2.64 ± 0.63 and total serum bilirubin estimated on 3^rd^ day of life was 16.14 ± 1.4. 3^rd^ day serum bilirubin and cord blood bilirubin were correlated positively with the (r=0.087). Umbilical cord blood bilirubin estimation is a non-invasive, economical, and simple method of predicting subsequent neonatal hyper-bilirubinemia that can help clinicians for early discharge of normal neonates and follow-up in high-risk infants.

## Background:

Jaundice is a common reason for neonatal therapy and is caused by a transitory decrease in the liver's conjugation ability. Serum total bilirubin within the physiological range indicates a balance in bilirubin excretion and synthesis in newborns. However, in certain newborns, these two processes are out of balance, resulting in hyper-bilirubinemia [1]. Nearly 80% of preterm newborns and 60% of term neonates have hyper-bilirubinemia, which has been linked to neuronal impairment that affects quality of life. In the Indian healthcare system, there is a trend of early release of healthy full-term babies due to economic considerations and a lack of resources [2]. Many of these newborns are readmitted to the NICU for hyper-bilirubinemia treatment, which necessitates phototherapy, adding to the cost burden on families and treating institutions. These readmissions expose healthy newborns to extremely infectious hospital surroundings, cause emotional disruptions in mothers and interfere with regular nursing habits [3]. Before releasing the new-born, the risk of substantial hyper-bilirubinemia is constantly assessed, including transcutaneous bilirubin, clinical assessment of risk factors and total blood bilirubin. Neonates released within 48 hours of delivery must be seen by a paediatrician within 2-3 days, according to American Academy of Paediatrics) [4]. A constant effort is made by Paediatricians to identification of newborns that are likely to develop jaundice. Different tools have been tried to evaluate the risk of significant hyperbilirubinemia including assessment of transcutaneous bilirubin, clinical assessment of risk factors and evaluation of total serum bilirubin before discharging the neonate [5]. This is slightly difficult to follow neonates in the Indian context owing to the scarcity of follow-up facilities, hence posing a challenge to paediatricians for the identification of neonates at hyper-bilirubinemia risk. Therefore, it is of interest to assess if cord blood bilirubin at birth can be used as a predictor of significant neonatal hyper-bilirubinemia needing phototherapy in full-term neonates.

## Materials and Methods:

The present prospective clinical study was conducted at Nandkumar Singh Chouhan Government Medical College, Khandwa and Madhya Pradesh. Verbal and written informed consent was taken from all the subjects before participation. The study included healthy term neonates from 37-42 weeks of gestation as assessed using the New Ballard score, neonates from both genders that were delivered by either cesarean or vaginal delivery with birth weight of ≥2500 grams. The exclusion criteria for the study were subjects with gestational age <37 weeks, low birth weight babies, perinatal hypoxia, sepsis and subjects with major congenital anomalies. Also, a 2ml cord blood sample was collected from all the newborns from the placental side of the cord during delivery in a red color-coded vacutainer. IT was ensured that samples were not exposed to the light while transporting these samples to the laboratory and during their storage and processing. Hemolyzed blood samples were excluded and bilirubin was evaluated using the Daizo method. Total serum bilirubin measurement was repeated on 3^rd^ day with serum samples obtained by venipuncture procedure. The study included 220 neonates with 110 subjects with hyper-bilirubinemia needing phototherapy as cases and 110 neonates without hyper-bilirubinemia as controls. The data gathered were analyzed statistically using SPSS software version 24.0 for assessment of descriptive measures, Student t-test, ANOVA, Pearson correlation coefficient, and Chi-square test. The results were expressed as mean and standard deviation and frequency and percentages. The p-value of <0.05 was considered.

## Results:

There were 56% (n=112) males and 44% (n=88) females in the present study. 110 neonates had hyper-bilirubinemia and needed phototherapy and 110 neonates had no hyper-bilirubinemia. Rh incompatibility was seen in 14% (n=14) subjects and ABO incompatibility was seen in 6% (n=6) subjects. The mean APGAR score in study subjects was 7.82 and the mean birth weight was 2.37 kg as shown in Table 1. The study results showed that for comparison of umbilical cord bilirubin (UCB) and 3^rd^-day bilirubin levels in neonates requiring phototherapy, in subjects requiring phototherapy, the mean value of umbilical cord blood was 2.64 ± 0.656 and 3^rd^-day total bilirubin was 16.14±1.681 showing statistically significant results with p=0.001. In subjects that did not need phototherapy, the mean umbilical cord blood value was 1.64 ± 0.511, and 3^rd^-day total bilirubin was 9.90 ± 2.057. The difference was highly statistically significant with p=0.001. It was seen that for assessment of the correlation of UCB and 3^rd^-day TB in study subjects, the r- value in subjects needing phototherapy was 0.085, and in subjects that did not need phototherapy, r- value was-0.023. The results were statistically non-significant for both I n subjects that needed or did not need phototherapy with p=0.546 and 0.863 respectively Table 1. It was also seen that for specificity and sensitivity of 3^rd^ day total bilirubin and umbilical cord bilirubin in prediction of hyper-bilirubinemia, for UCB, were shown in Table 2.

## Discussion:

The present study assessed cord blood bilirubin in 220 neonates immediately following delivery that was followed for the next 3 days and serum total bilirubin was assessed on the 3^rd^ day of life. There were 56% (n=112) males and 44% (n=88) females in the present study. 110 neonates had hyperbilirubinemia and needed phototherapy and 110 neonates had no hyperbilirubinemia. Rh incompatibility was seen in 14% (n=14) subjects and ABO incompatibility was seen in 6% (n=6) subjects these findings were similar to the Anjanappa *et al.* in 2023 [6] and Bhat *et al.* in 2019 [7]. The mean APGAR score in study subjects was 7.82 and the mean birth weight was 2.37 kg. These data were comparable to the studies of Rehna *et al.* [8] in 2012 and Reddy *et al.* [9] in 2021 where authors assessed subjects with demographic data and neonatal hyperbilirubinemia similar to the present study in their respective studies. In the subjects requiring phototherapy, the mean value of umbilical cord blood was 2.64 ± 0.656 and 3^rd^-day total bilirubin was 16.14 ± 1.681 [statistically significant p=0.001]. In subjects that did not need phototherapy, the mean umbilical cord blood value was 1.64 ± 0.511 and 3^rd^ day total bilirubin was 9.90 ± 2.057 [highly statistically significant p=0.001]. These results were consistent with the findings of Kumar *et al.* [10] in 2005 and Vasudevan *et al.* [11] in 2013 where a comparison of umbilical cord bilirubin (UCB) and 3^rd^-day bilirubin levels in neonates requiring phototherapy reported by the authors in their studies had results similar to the present study. For both groups, the association between UCB and 3^rd^ day TB in study participants was statistically non-significant (p=0.546 and 0.863, respectively). These findings were comparable to the findings of Gupta *et al.* in 2021 [12] and Gilbert *et al.* [13] in 2023. Similar findings to the current study were previously published by Zeitoun *et al.* [4] in 2013 and Bernaldo *et al.* [14] in 2004 for the evaluation of the association between UCB and 3^rd^ day TB in the study participants.

## Conclusion:

A cut-off value of 2.5mg/dL in cord blood bilirubin can be used for the prediction of significant neonatal hyper-bilirubinemia needing phototherapy in full-term neonates. Also, umbilical cord blood bilirubin estimation is a non-invasive, economical, and simple method of prediction of subsequent neonatal hyper-bilirubinemia that can help clinicians for early discharge of normal neonates and follow-up in high-risk infants.

## Figures and Tables

**Table 1:** Baseline characteristics, comparison and correlation of umbilical cord bilirubin (UCB) and 3^rd^-day bilirubin levels in neonates requiring phototherapy

**Characteristics**		**N**		**%**	
**Gender**					
Males		112		56	
Females		88		44	
Neonates with no hyper-bilirubinemia		110		100	
Neonates with hyper-bilirubinemia need phototherapy		80		80	
Rh incompatibility		14		14	
ABO incompatibility		6		6	
Average APGAR score		7.82			
Average birth weight (kg)		2.37			
**Phototherapy**	**Total bilirubin**	**Mean**	**r-value**		**p-value**
Yes	UCB	2.64±0.656	0.085		0.546
	3^rd^ day TB	16.14±1.681			
No	UCB	1.66±0.511	-0.023		0.863
	3^rd^ day TB	9.90±2.057			

**Table 2:** Specificity and sensitivity of 3^rd^ day total bilirubin and umbilical cord bilirubin in prediction of hyper-bilirubinemia

**Test result variables**	**Positive if ≥**	**Sensitivity**	**Specificity**
UCB	0	100	0
	1.5	100	32
	2.5	54	96
	3.5	10	100
	5	0	100
Day 3 total bilirubin	1	100	0
	5.5	100	4
	9.5	100	40
	10.5	100	70
	11.5	100	72
	12.5	94	98
	13.5	86	100
	14.5	84	100
	15.5	76	100
	16.5	40	100
	17.5	20	100
	18.5	4	100
	20	0	100
